# Socioeconomic factors and lifestyles influencing the incidence of calcaneal fractures, a national population-based survey in China

**DOI:** 10.1186/s13018-019-1493-2

**Published:** 2019-12-10

**Authors:** Yanbin Zhu, Jia Li, Song Liu, Wei Chen, Lin Wang, Xiaolin Zhang, Yingze Zhang

**Affiliations:** 1 Department of Orthopaedic Surgery, The Third Hospital of Hebei Medical University, NO.139 Ziqiang Road, Shijiazhuang, Hebei 050051 People’s Republic of China; 2 Key laboratory of biomechanics of Hebei Province, Shijiazhuang, Hebei 050051 People’s Republic of China; 3 Chinese Academy of Engineering, Beijing, 100088 People’s Republic of China; 4 Department of Orthopaedic Surgery, The First Hospital of Hebei Medical University, Shijiazhuang, Hebei 050031 People’s Republic of China; 5 Department of statistics and epidemiology, Hebei Medical University, Shijiazhuang, Hebei 050000 People’s Republic of China

**Keywords:** Calcaneal fracture, Epidemiology, Population-based, Risk factor, China

## Abstract

**Background:**

We aimed to do a national survey on the population-based incidence of calcaneal fracture in China.

**Methods:**

All the data on calcaneal fractures were available from the China National Fracture Survey (CNFS) between January and May in 2015. And in the CNFS, all eligible household members were sampled from 8 provinces, 24 urban cities and 24 rural counties in China, using stratified random sampling and the probability proportional to size method. Questionnaires were sent to every participant for data collection and quality control was accomplished by our research team members.

**Results:**

A total of 512187 valid questionnaires were collected and relevant data were abstracted and analyzed. There were 59 patients with 62 calcaneal fractures occurring in 2014, indicating that the incidence was 11.5/100,000 person-years, 17.3/100,000 in males, and 5.5/100,000 in females. BMI ≥ 28.0 kg/m^2^, scarce meat consumption, smoking, alcohol consumption, average sleep time < 7 h/day, and previous history of fracture were identified as independent risk factors for calcaneal fracture.

**Conclusions:**

Specific public health policies focusing on quitting smoking, decreasing alcohol consumption, and encouraging individuals to obtain sufficient sleep should be implemented. Reasonable meat consumption and maintaining a normal body weight should be emphasized in individuals, especially in those with previous fracture.

## Introduction

Calcaneal fracture was firstly described by Norris in the year 1839 and then was named by Malgaigne using his name in the year 1843 [1, 2]. Calcaneal fracture was one of the common injuries in the department of emergency and orthopedics, and accounting for 60–78% of fractures in tarsal, 30% in foot, and 2–3.1% of all fractures [3–5]. As far as we know, only two studies were conducted to report the population-based incidence of calcaneal fracture [5, 6]. In the former one [5], authors used the Finland National Hospital Discharge Register to assess the epidemiology of calcaneal fractures and reported the incidence of 12.5 in males and 3.9 in females per 100,000 persons, respectively. However, only hospitalized patients selected as study objects inevitably result in the underestimation of incidence rate of calcaneal fractures. In the latter one from Edinburgh [6], authors reported the annual incidence of calcaneal fracture was 16.5 and 6.3 per 100,000 in males and females, and concluded males were more likely to sustain this injury relative to females. But the methodological design as single hospital might compromise the results, although the sample size was not small (752 fractures in 697 patients). Another Finnish study presented rising incidence of low-trauma foot and calcaneus fractures among adults > 50 years in 1970–2005, but did not analyze the incidence of calcaneal fractures alone [7]. However, extrapolation of conclusions obtained from these studies to our population and implementation in our routine clinical evaluation might not be valid due to differences in ethnic groups, economic development, culture practice, and lifestyles among different countries. Furthermore, we have not found any previous studies that aimed to investigate the socioeconomic risk factors associated with calcaneal fractures.

Currently, the National Health Services Survey (NHSS) is the epidemiologic database of the national level in China for the collection of data on self-reported fractures at 2 weeks before the surgery. However, we were unable to obtain any information on less severe fractures treated by conservative methods. Furthermore, this national survey system only collected the most basic data on fractures (e.g., age, gender, and fracture occurrence timing), but without any information on type of fracture, body site, injury mechanisms, and related potential risk factor (socioeconomic and lifestyles).

Given above, we designed and performed the China National Fracture Study (CNFS) in 2015 with aims to investigate the population-based incidence of traumatic fracture of the trunk, arms, or legs and associated socioeconomic risk factors and lifestyles. The overall results have been published recently [8]. In this study, we extracted related data on calcaneal fractures from the CNFS database and aimed (1) to report the national population-based incidence of calcaneal fracture in China and (2) to explore the associated risk factors in term of demographics, socioeconomics, and lifestyles.

## Methods

### Sampling method

The entire sampling process of CNFS was completed with combined use of optimum allocation and random stratified and probability proportionate to size (PPS) sampling method. During the first phase, 8 provinces (municipalities) were initially selected from 31 provinces (municipalities or autonomous regions) in mainland China based on socioeconomic development and climate, using stratified random sampling method. And within each targeted province (municipalities), sampling was done separately in urban and rural areas (Table 1).

**Table 1:** National incidence of calcaneal fractures among Chinese population by demographic, socioeconomic, and geographic factors in 2014

Items	Sample size	Male		Female		Total	
		Case	Incidence (1/100000)	Case	Incidence (1/100000)	Case	Incidence (1/100000)
Overall	512187	45	17.3 (12.3–22.4)	14	5.5 (2.6–8.4)	59	11.5 (8.6–14.5)
Age (years)							
0–14	81166	4	9	1	2.7	5	6.2
			(0.2–17.9)				(0.8–11.6)
15–44	236206	18	15.2	4	3.4	22	9.3
			(8.2–22.3)		(0.1–6.7)		(5.4–13.2)
45–64	138533	19	27.5	5	7.2	24	17.3
			(15.1–39.8)		(0.9–13.5)		(10.4–24.3)
65 +	56282	4	14.2	4	14.2	8	14.2
			(0.3–28.2)		(0.3–28.1)		(4.4–24.1)
*p* value for trend test	512187	0.13		0.028		0.021	
Ethnicity							
Han	477508	43	17.8	14	5.9	57	11.9
			(12.5–23.1)		(2.8–9.1)		(8.8–15)
Others	34679	2	11.4	0	0	2	5.8
			(− 4.4–27.1)				
*p* value for difference test	512187	0.533		0.314		0.437	
Region							
East	232998	24	20.1	7	6.2	31	13.3
			(12.1–28.1)		(1.6–10.7)		(8.6–18)
Central	99109	7	14.1	3	6.1	10	10.1
			(3.6–24.5)				(3.8–16.3)
West	180080	14	15.5	4	4.5	18	10
			(7.4–23.6)		(0.1–8.8)		(5.4–14.6)
*p* value for difference test	512187	0.602		0.863		0.553	
Urbanization							
Urban area	203101	12	11.7	6	6	18	8.9
			(5.1–18.3)		(1.2–10.8)		(4.8–13)
Rural area	309086	33	21	8	5.3	41	13.3
			(13.8–28.2)		(1.6–8.9)		(9.2–17.3)
*p* value for difference test	512187	0.078		0.814		0.151	
Education							
Illiterate	74937	9	26.1	7	17.3	16	21.4
			(9.1–43.2)		(4.5–30.1)		(10.9–31.8)
Primary school	158970	24	29.9	2	2.5	26	16.4
			(17.9–41.9)				(10.1–22.6)
Junior high school	121415	7	11.4	4	6.7	11	9.1
			(3–19.8)		(0.1–13.2)		(3.7–14.4)
Senior high school or above	40841	4	18.5	0	0	4	9.8
			(0.4–36.7)				(0.2–19.4)
*p* value for trend test	396163	0.104		0.036		0.023	

For urban areas, using the optimum allocation and random stratified and probability proportional to size method, we selected a certain number of streets ranging from one to six in each sampled city and a certain number ranging from one to ten neighborhood communities from each chosen street based on the geographical location from west to east on the electronic map. The total number of families in each neighborhood community was determined by the average number of household members according to the latest official census data in China. All members of eligible families to be invited to participate in this study must have lived in their current residence for at least 6 months.

For rural areas, we sampled 1–5 counties in each selected province and then in each county, 1–8 towns were selected. In each town, 1–14 administrative villages were sampled. The sampling process was completed using the probability proportional to size method. In each village, households were calculated and selected based on probability proportional to size principles. Similarly, as in urban areas, all members of eligible families to be invited to participate in this study must have lived in their current residence for at least 6 months.

### Participants and survey

In principle, eligible household members must be personally interviewed by trained research team members. However, for preschool and primary school children, their information should be provided by their guardians in order to ensure data accuracy. For participants who remained non-contactable after repeated visits, telephone surveys had to be used. For any member in selected household who refused to participate, an alternative household was randomly selected from the candidate list.

A standardized questionnaire was administered by our trained research team for data collection. The detailed information included age, sex, Chinese ethnic nationality, marital status, residence, income status, occupation, lifestyles (smoking, alcohol drinking, tea, coffee, and carbonated beverages and daily consumption of meat, protein product, and dairy products) for all participants, age of menopause, and the number of births for women. Individuals who had calcaneal fractures between January 1 and December 31, 2014, then must answer a more detailed accessory questionnaire regarding the fracture occurrence, date and place, and injury mechanism. In addition, they were asked to provide medical records of the index injury, including radiographs, diagnostic reports, and medical reports. And if these data were not available, the survey team paid for individual participants to obtain a new radiograph of their reported calcaneus at a local hospital for reappraisal.

Eight quality control teams were established (one per province) to check for the quality of related data collection. The CNFS was approved by the Institutional Review Board of the 3rd Hospital of Hebei Medical University and written informed consent was obtained from each participant before data collection.

### Definition of variables of interest

Individuals were divided into Han ethnicity and others including all the national minority ethnicity. The body mass index (BMI) was calculated as weight divided by the square of height, and was grouped based the reference criteria suited to Chinese people: underweight, < 18.5; normal, 18.5–23.9; overweight, 24–27.9; obesity, > = 28 [8, 9]. Daily diet and drinking including meat and products, bean products, milk and dairy products, coffee, tea, and carbonated beverages were divided into 5 groups based on the frequency of consumption: never, always (at least 1 per day), often (1/day–1/week), occasionally (1/week–1/month), and seldom (< 1/month). Calcium or vitamin D supplement was defined as positive if participants acknowledged they received calcium or vitamin D or both related medicine or nourishment at least 1 month before the calcaneal fracture occurrence. Urbanization was divided into 2 groups: (1) rural area (village) and (2) urban areas (cities of levels).

### Statistical analysis

Incidence rates for calcaneal fractures were estimated for the overall population and for subgroups such as age, ethnics, region, education level, and urbanization level, stratified by gender. For unordered categorical variables such as region, urbanization, and ethnics, the chi-square test was used to test the difference. For ordered categorical variables such as age and education level, we entered the related data as a continuous variable into a univariate logistic regression model to assess the incidence trend.

Case group was defined as adult patients sustaining calcaneal fractures in 2014, and control group was defined as adult individuals without any fracture in 2014. Chi-square test was used to investigate the potential correlations between calcaneal fractures and various factors of interest. Finally, multivariate logistic regression models were used to explore the independent risk factors associated with calcaneal fractures. *P* < 0.05 was set as the statistical significance level. Odd ratio (OR) values and corresponding 95% confidence interval (95% CI) were used to indicate the strength of correlation of risk factor. The Hosmer–Lemeshow test was used to examine goodness-of-fit of the final model and a *p* value > 0.05 indicated an acceptable fitness. SPSS 19.0 was used to perform all the analyses (SPSS Inc., Chicago, IL, USA).

## Results

During the survey, a total of 512,187 valid questionnaires were collected and relevant data were abstracted and analyzed. Through the year 2014, 1763 patients sustained traumatic fractures (1833 fractures). Of them, there were 59 patients with 62 calcaneal fractures, indicating that the incidence rate was 11.5 (95%CI, 8.6–14.5) per 100,000 person-years. There were 45 males and their median age was 45 years and the corresponding incidence rate was 17.3 (95%CI, 12.3–22.4) per 100,000 person-years; there were 14 females sustaining calcaneal fractures with median age of 60 years, and the corresponding incidence was 5.5 (95%CI, 2.6–8.4) per 100,000 person-years.

Fall from a height was the most common cause, leading to 47.5% (28/59) of calcaneal factures, and was followed by slip, trip, or fall from standing height, chairs, or stairs (42.4%, 25/59); traffic accidents (5.1%, 3/59); and crushing injuries (5.1%, 3/59) (Table 2). In the middle-aged and elderly patients, 60% (15/25) of the injuries were caused by low-energy slip, trip, or fall from standing height, chairs, or stairs. In terms of occurrence place of calcaneal fracture, home and building site were the first two common sites which accounted for 71.2% of the overall injuries. Besides, work unit was also an important place that should be considered, where 15.3% (9/59) of calcaneal fractures happened (Table 3).

**Table 2:** The causal mechanisms for calcaneal fractures in China in 2014 (*n*, %)*

Injury mechanism	Children	Adult (≥ 15 years)		Total
	(0–14 years)	Male	Female	
Fall from heights	2 (66.7)	13 (41.9)	13 (52.0)	28 (47.5)
Slip, trip, or fall from standing height, chairs, or stairs	1 (33.3)	16 (51.6)	8 (32.0)	25 (42.4)
Traffic accident	0	0	3 (12.0)	3 (5.1)
Crushing injury	0	2 (6.5)	1 (4.0)	3 (5.1)
Sum	3 (5.1)	31 (52.5)	25 (42.4)	59 (100.0)

**Table 3:** The place of calcaneal fracture occurrence in 2014 (*n*, %)*

Place of fracture occurrence	Children	Adult (≥ 15 years)		Total
		Male	Female	
Home	3 (60.0)	12 (29.3)	8 (61.5)	23 (39.0)
Work unit	1 (20.0)	7 (17.1)	1 (7.7)	9 (15.3)
Building site	0	17 (41.5)	2 (15.4)	19 (32.2)
Road	0	1 (2.4)	2 (15.4)	3 (5.1)
Expressway	0	2 (4.9)	0	2 (3.4)
School	1 (20.0)	1 (2.4)	0	2 (3.4)
Others	0	1 (2.4)	0	1 (1.7)
Sum	5 (8.5)	41 (69.5)	13 (22.0)	59 (100.0)

Table 1 presented the population-based incidence rates of calcaneal fractures in overall populations and subgroups based on democratic and socioeconomic characteristics, stratified by gender. There was no significant difference in incidence between those of Han ethnicity and all other ethnicities combined, nor was there any significant difference according to geographical region, urbanization, or education, either for overall population or any gender (Table 1). Stratified by age, males of 45–64 years and females of ≥ 65 years had the highest incidence rate (27.5 and 14.2 per 100,000 person-years), respectively. The trend difference of incidence rate by age in females and overall population approach to significance (*p* = 0.028; *p* = 0.021), but was non-significant in males (*p* = 0.130). Stratified by education level, the illiterate in females and males with primary school level had the highest incidence in respective subgroup, and the trend difference test demonstrated the significant result in females (*p* = 0.036) but not in males (*p* = 0.104)

Table 4 presented the detailed results of univariate chi-square test between case and control group in adults (≥ 15 years). We could find that there were significant differences between calcaneal fractures and controls in term of gender (*p* < 0.001), BMI (*p* = 0.015), education level (*p* = 0.038), occupation (*p* = 0.032), meat and product (*p* < 0.001), smoking status (*p* < 0.001), alcohol consumption (*p* < 0.001), sleep time per day (*p* < 0.001), calcium or vitamin D supplement or both (*p* = 0.025), and history of fracture (*p* < 0.001). And in other variables, we did not observe the significant differences, such as age and region.

**Table 4:** Detailed results of univariate analysis for variables of interest

Variables	Case, *n* = 54 (%)	Control, *n* = 429,375(%)	*p*
Gender			< 0.001
Male	41 (75.9)	214,501 (50)	
Female	13 (24.1)	214,874 (50)	
Age (year)			
15–44	22 (40.7)	235,657 (54.9)	0.101
45–64	24 (44.4)	137,779 (32.1)	
≥ 65	8 (14.8)	55,939 (13)	
Region			0.728
Eastern	27 (50)	193,223 (45)	
Middle	9 (16.7)	85,630 (19.9)	
Western	18 (33.3)	150,522 (35.1)	
Urbanization			0.213
Rural area	17 (31.5)	258,563 (60.2)	
Urban area	37 (68.5)	170,812 (39.8)	
Ethnicity			0.158
Han	53 (98.1)	400,874 (93.4)	
Other	1 (1.9)	28,501 (6.6)	
BMI			0.015
18.5–23.9	27 (50)	282,433 (65.8)	
24–27.9	18 (33.3)	102,964 (24)	
≥ 28	6 (11.1)	17,730 (4.1)	
< 18.5	3 (5.6)	26,248 (6.1)	
Education			0.038
Illiterate	16 (29.6)	74,774 (17.4)	
Primary school	23 (42.6)	162,924 (37.9)	
Junior high school	11 (20.4)	134,891 (31.4)	
Senior high school or above	4 (7.4)	56,786 (13.2)	
Occupation			0.032
Unemployed	1 (1.9)	32,590 (7.6)	
Office worker	2 (3.7)	61,747 (14.4)	
Manual worker	25 (46.3)	148,165 (34.5)	
Farmer	19 (35.2)	105,960 (24.7)	
Retired	4 (7.4)	30,197 (7)	
Students	1 (1.9)	34,833 (8.1)	
Other	2 (3.7)	15,883 (3.7)	
Meat and product			
Never or seldom	9 (16.7)	22,000 (5.1)	< 0.001
Always	28 (51.9)	216,500 (50.4)	
Often	9 (16.7)	130,155 (30.3)	
Occasionally	8 (14.8)	60,720 (14.1)	
Seldom	9 (16.7)	19,448 (4.5)	
Dairy and product			0.944
Never	29 (53.7)	210,279 (49.0)	
Always	9 (16.7)	69,907 (16.3)	
Often	8 (14.8)	76,218 (17.8)	
Occasionally	8 (14.8)	72,971 (17)	
Bean product			0.099
Never or seldom	7 (13.0)	47,925 (11.1)	
Always	13 (24.1)	80,682 (18.8)	
Often	16 (29.6)	200,433 (46.7)	
Occasionally	18 (33.3)	100,335 (23.4)	
Alcohol consumption			< 0.001
No	19 (35.2)	289,344 (67.4)	
Yes	35 (64.8)	140,031 (32.6)	
Cigarette smoking			< 0.001
No	24 (44.4)	324,652 (75.6)	
Yes	30 (55.6)	104,723 (24.4)	
Carbonate beverages			0.265
Never or seldom	33 (61.1)	310,164 (72.3)	
Always	1 (1.9)	4766 (1.1)	
Often	3 (5.6)	58,481 (13.6)	
Occasionally	6 (11.1)	55,964 (13)	
Coffee			0.160
No	53 (98.1)	401,055 (93.4)	
Yes	1 (1.9)	28,320 (6.6)	
Tea			0.093
Never or seldom	23 (42.6)	255,980 (59.6)	
Always	18 (33.3)	103,425 (24.1)	
Often	8 (14.8)	41,056 (9.6)	
Occasionally	5 (9.3)	28,914 (6.7)	
Living circumstance			0.523
Single-storey house	24 (44.4)	170,315 (39.7)	
House ≤ 7 storey	28 (51.9)	227,535 (53)	
House > 7 storey	2 (3.7)	31,525 (7.3)	
Calcium or vitamin D supplement or both			0.025
No	47 (87)	404,323 (94.2)	
Yes	7 (13)	25,052 (5.8)	
Average sleep time (hours) per day			< 0.001
≥ 7	20 (37)	280,212 (65.3)	
< 7	34 (63)	149,163 (34.7)	
Previous history of fracture			< 0.001
No	48 (88.9)	419,666 (97.7)	
Yes	6 (11.1)	9709 (2.3)	

Table 5 summarized independent risk factors for calcaneal fractures in adults, after adjustment for other confounding variables. BMI ≥ 28.0 significantly increased the risk of calcaneal fracture by 3.04 (95% CI, 1.25–7.38) times, compared to the normal BMI (18.5–23.9). Patients with previous history of fracture had an increased risk of calcaneal fracture by 3.58 times (95% CI, 1.52–8.43). And compared to those having enough sleep time (≥ 7 h/day), average sleep time < 7 h/day increased the risk of calcaneal fracture by 2.85 times (95%CI, 1.63–4.95). In addition, bad lifestyles such as smoking and alcohol consumption were identified as significant risk factors and increased the 2.54 and 2.51-time risk of calcaneal fractures, respectively.

**Table 5:** Results of multivariate logistic regression of risk factors for calcaneal fractures

Variables	Exp (B)	95%CI		*p*
		Lower limit	Upper limit	
BMI				
18.5–23.9	Reference			
< 18.5	1.45	0.44	4.77	0.546
24–27.9	1.56	0.86	2.84	0.144
≥ 28.0	3.04	1.25	7.38	0.014
Meat and product consumption				
Always	Reference			
Often	0.529	0.250	1.122	0.097
Occasionally	1.022	0.465	2.245	0.957
Seldom or never	3.182	1.494	6.779	0.003
Smoking	2.54	1.41	4.58	0.002
Alcohol consumption	2.51	1.36	4.64	0.003
Previous fracture history	3.58	1.52	8.43	0.003
Sleep < 7 h/day	2.85	1.63	4.95	<0.001

In the final multivariate logistic regression model, the Hosmer–Lemeshow test demonstrated the adequate fitness (X^2^ = 3.253, *P* = 0.917).

## Discussion

Currently, optimal management of calcaneal fractures is controversial [10], and compartment syndrome of foot is one of the most important implications after injury, with high risk of morbidity and poor outcome, including persistent neurologic deficits or amputation [11]. Extensive differences in the epidemiological characteristics of calcaneal fractures among geographic regions, ethnic groups, races, and gender have been reported [5–7, 12], and the results should be for references only but not be directly applied in Chinese policy makers and clinical practices. In the present study, we used the data from CNFS database to resolve this issue and the results showed that the incidence rate of calcaneal fracture was 11.5/100,000 person-years with 17.3/100,000 person-years in males and 5.5/100,000 person-years in females. Fall from height remained in the first place in the causes of calcaneal fracture occurrence, closely followed by skip, trip, or fall from chair or stairs. Over 70% of the injuries occurred at home and building site. In adults, BMI ≥ 28.0, seldom or no consumption of meat and product, smoking, alcohol consumption, average sleep time < 7 h/day, and previous history of fracture significantly increased the risk of calcaneal fractures.

The incidence rate of calcaneal fractures reported in this study was consistent with that in a UK study [6] but slightly higher than the study in Finland [5]. The former one reported the incidence of 11.5 per 100,000 for overall population, 16.5/100,000/year in males and 6.26/100,000/year in females, which were all similar as ours [6]. However, regarding the injury mechanism, the authors reported falls from a height (71.5%) as the first place, and 64.3% of these were from 6 ft and above, which was considerably higher than ours (47.5%). We thought this great gap mainly lied in the patient source in their study, all patients were from a level-1 trauma center and presented with more severe injuries necessitating hospitalization and operation. In contrast, in our study, patients were sampled based on overall population and therefore were more representative. In fact, results in this study showed that a certain proportion (60%, 15/25) of patients were caused by low-energy slip or fall from low height such as chair, bed, or stairs, in the elderly In the latter study [5], authors reported the slightly lower incidence rate (12.5/100,000 in males and 3.9/100,000 in females) of hospitalization patients in adults and concluded the incidence remained relatively stable during the past 26 years. In addition, authors observed that male patients with calcaneal fractures were much younger than females (43 vs 61), which was similar as ours (median, 45 years in males and 60 years in females). We infer that this might be related to the high-energy activities in males and relatively poorer bone quality and mass in females.

In the current studies, unhealthy lifestyles or bad habits such as smoking, alcohol consumption, and sleeping time less than 7 h per day was identified as independent risk factor for calcaneal fractures in adults. Alcohol consumption as a risk factor for traumatic fracture had been well recognized in the literature [13, 14]. Scholes et al. suggested consuming more than 8 units of alcohol for men or more than 6 units for women in the past week increased the 1.65-time and 2.07-time risk of fractures in individuals ≥ 55 years [13]. The underlying mechanism might be related to metabolic effects and alcohol-related falls [13, 14]. Stone et al. [15] reported that women who slept for 5 h or less or 5–7 h had the higher risk of frequent falls, compared to those with adequate sleep (7–8 h/day). And Holmberg et al. [16] got similar findings in males that sleep disturbances contributed to the increased risk in most fractures. Tobacco consumption was identified to have a significant negative effect on bone mineral density and is a risk factor for fractures in general [17, 18]. Cornuz et al. [17] did a large study of 116 229 female aged 34–59 years with up to 12-year follow-up and found an increased relative risk of 1.3 (95% CI 1.0–1.7) for hip fractures in current smokers when compared with non-smokers. A Finland study including 11,798 women aged 47–56 years with follow-up of 5 years showed smokers with less than 20 cigarettes per day and ≥ 20 cigarettes per day increased the 1.73-time and 2.94-time risk of ankle fractures, respectively [19]. In addition, Cornuz et al. suggested that quitting smoking could at least reduce 30% of the fractures, at 10-year follow-up after quitting [17]. Therefore, healthy lifestyles like decreased alcohol consumption, non-smoking or quitting smoking, and adequate sleep duration should clearly be advocated to reduce fracture risk.

Obesity (BMI≥28.0) was identified as a significant risk factor for calcaneal fracture and compared to those with normal BMI (18.5–23.9), obese individuals had the 3-time risk of calcaneal fracture. We suggested that heavy weight plays an important role in calcaneal fracture occurrence, which brought greater press on calcaneus when moving or falls from height or standing height, especially in young and middle-aged patients. Nielson et al. and Compston et al. suggested the reduced physical mobility and repeated falls were at least partially responsible for fractures in elderly patients with decreased bone mass [20, 21]. In addition, obesity-related chronic morbidities as diabetes mellitus and cerebrovascular diseases predisposed to the fractures [20]. History of previous fracture as a risk factor for the subsequent fracture had been well identified in literature and was re-identified in this study. In a meta-analysis by Klotzbuecher et al. [22], authors concluded history of prior fracture at any site was an important risk factor for future fractures and the risk of future fractures appeared to increase with the number of prior fractures. Kanis et al. drew the similar conclusion in a meta-analysis that previous history of fracture conferred an increased 1.86-time risk of subsequent fracture of any site beyond that explanation by measurement of BMD [23]. Reasonable diet with balance portion of meat and vegetables was necessitated for bone health, and meat consumption < 1 time/month was identified as a risk factor for calcaneal fracture. The underlying mechanism might be related to the animal protein and minerals in meat that were necessary to human, but remains to be investigated.

This is currently the largest questionnaire survey of incidence and risk factors for calcaneal fractures. Despite this, some potential limitations must be mentioned. Firstly, the retrospective nature of this study had its intrinsic weakness in accuracy of collected data, which might incur recall biases. Secondly, the results of patients’ self-report on fracture and individual life styles might be affected due to manners or customs. Thirdly, the incidence rate of calcaneal fracture could be underestimated, as we could not capture data on the individual who had died in this index injury or coexisting diseases or complications.

In summary, the current study provided detailed information about the national population-based incidence, characteristics, and related risk factors of calcaneal fractures, which could be used as reference data for healthcare policy makers and health consultation and prevention for individuals. Specific public health policies focusing on decreasing alcohol consumption, quitting smoking, and encouraging individuals to obtain sufficient sleep should be implemented. Reasonable meat consumption and maintaining a normal body weight should be emphasized in individuals, especially in those with history of previous history.

## Data Availability

The datasets generated and/or analyzed during the current study are not in public but are available from the corresponding author on reasonable request.

## Abbreviations

BMD: Bone mineral density
BMI: Body mass index
CNFS: China national fracture study
NHSS: National health services survey
OR: Odd ratio
PPS: Probability proportionate to size

## References

[1] Swanson SA, Clare MP, Sanders RW (2008). Management of intra-articular fractures of the calcaneus. Foot Ankle Clin.

[2] Coughlin MJ, Mann RA, Saltzman CL. Surgery of the foot and ankle. 8th ed, 2008; pp 461-462.

[3] Zhang Y. Clinical epidemiology of orthopaedic trauma, 2 ed., Thieme, Stuttgart, 2012.

[4] Cohen Zvi, Volpin Gershon, Shtarker Haim (2011). Surgical Treatment of Displaced Calcaneal Fractures. European Instructional Lectures.

[5] Haapasalo H, Laine HJ, Mäenpää H, Wretenberg P, Kannus P (2017). Epidemiology of calcaneal fractures in Finland. Foot Ankle Surg.

[6] Mitchell MJ, Mckinley JC, Robinson CM (2009). The epidemiology of calcaneal fractures. Foot.

[7] Kannus P, Niemi S, Palvanen M, Sievänen H, Parkkari J (2008). Rising incidence of low-trauma fractures of the calcaneus and foot among Finnish older adults. J Gerontol A Biol Sci Med Sci.

[8] Chen W, Lv H, Liu S, Liu B, Zhu Y (2017). National incidence of traumatic fractures in China: a retrospective survey of 512 187 individuals. Lancet Glob Health.

[9] Zhu Y, Liu S, Zhang X, Chen W, Zhang Y (2017). Incidence and risks for surgical site infection after adult tibial plateau fractures treated by ORIF: a prospective multicentre study. Int Wound J.

[10] Gougoulias N, Khanna A, McBride DJ, Maffulli N (2009). Management of calcaneal fractures: systematic review of randomized trials. Br Med Bull.

[11] Lutter C, Schöffl V, Hotfiel T, Simon M, Maffulli N (2019). Compartment syndrome of the foot: an evidence-based review. J Foot Ankle Surg.

[12] Tadros AMA, Eid HO, Abu-Zidan FM (2010). Epidemiology of foot injury in a high-income developing country. Injury..

[13] Scholes S, Panesar S, Shelton NJ, Francis RM, Mirza S (2014). Epidemiology of lifetime fracture prevalence in England: a population study of adults aged 55 years and over. Age Ageing.

[14] Clark MK, Sowers MF, Dekordi F, Nichols S (2003). Bone mineral density and fractures among alcohol-dependent women in treatment and in recovery. Osteoporos Int.

[15] Stone KL, Ancoliisrael S, Blackwell T, Ensrud KE, Cauley JA (2008). Actigraphy-measured sleep characteristics and risk of falls in older women. Arch Intern Med.

[16] Holmberg AH, Johnell O, Nilsson PM, Nilsson J, Berglund G (2006). Risk factors for fragility fracture in middle age. A prospective population-based study of 33,000 men and women. Osteoporos Int.

[17] Cornuz J, Feskanich D, Willett WC, Colditz GA (1999). Smoking, smoking cessation, and risk of hip fracture in women. Am J Med.

[18] Law MR, Hackshaw AK (1997). A meta-analysis of cigarette smoking, bone mineral density and risk of hip fracture: recognition of a major effect. BMJ..

[19] Valtola A, Honkanen R, Kröger H, Tuppurainen M, Saarikoski S (2002). Lifestyle and other factors predict ankle fractures in perimenopausal women: a population-based prospective cohort study. Bone..

[20] Compston JE, Watts NB, Chapurlat R, Cooper C, Boonen S (2011). Obesity is not protective against fracture in postmenopausal women: GLOW. Am J Med.

[21] Nielson CM, Marshall LM, Adams AL, Leblanc ES, Cawthon PM (2011). BMI and fracture risk in older men: the osteoporotic fractures in men study (MrOS). J Bone Miner Res.

[22] Klotzbuecher CM, Ross PD, Landsman PB, Iii TAA, Berger M (2000). Patients with prior fractures have an increased risk of future fractures: a summary of the literature and statistical synthesis. J Bone Miner Res.

[23] Kanis JA, Johnell O, Laet CD, Johansson H, Oden A (2004). A meta-analysis of previous fracture and subsequent fracture risk. Bone.

